# Tumor‐derived Vimentin as a novel biomarker for distinct subtypes predicting adjuvant chemotherapy resistance and T‐cell‐inflamed phenotype in small cell lung cancer

**DOI:** 10.1002/mco2.370

**Published:** 2023-10-01

**Authors:** Chaoqiang Deng, Yue Wang, Fangqiu Fu, Di Li, Qiang Zheng, Yan Jin, Yuan Li, Haiquan Chen, Yang Zhang

**Affiliations:** ^1^ Department of Thoracic Surgery and State Key Laboratory of Genetic Engineering Fudan University Shanghai Cancer Center Shanghai China; ^2^ Institute of Thoracic Oncology Fudan University Shanghai China; ^3^ Department of Oncology Shanghai Medical College Fudan University Shanghai China; ^4^ Department of Pathology Fudan University Shanghai Cancer Center Shanghai China

**Keywords:** chemotherapy, immune landscape, long‐term survival, molecular subtype, SCLC

## Abstract

Despite recent progress in subtype classification for small cell lung carcinoma (SCLC), little is known about the biomarker for triple‐negative (ASCL1, NEUROD1, and POU2F3 negative) tumors. The long‐term survival, adjuvant chemotherapy (ACT) response, and immune milieu in different SCLC subtypes have also not been well established. Here, we retrospectively collected a large cohort of 192 primary SCLC tumors and reported that ASCL1‐, NEUROD1‐ and POU2F3‐dominant subtypes counted for 61.38%, 19.31%, and 6.21%, respectively. Subtype intra‐tumoral heterogeneity and co‐expression at the single‐cell level existed substantially. The expression of tumor‐derived Vimentin (VIM) was nearly restricted to triple‐negative SCLC tumors (15/19, 78.9%) while YAP1 expression was distributed widely in other subtypes. The SCLC subtyping model was independently prognostic of OS and RFS (*p* <  0.001 and *p* = 0.043). In particular, patients with ASCL1‐positive SCLC tumors can benefit more from ACT, and VIM‐positive tumors did the opposite. Compared with other subtypes, the VIM‐dominant SCLC subtype was associated with abundant but functionally impaired CD4^+^ and CD8^+^ T‐cells, which highly expressed inhibitory checkpoints and potentially benefit from PD‐L1 blockade therapy. Our study showed that tumor‐derived SCLC‐V subtype could independently predict ACT response. The distinct immune landscape between subtypes may help inform personalized immune therapeutic approaches.

## Graphical abstract

A large cohort of primary small‐cell lung cancer (SCLC) was collected to assess potential subtype markers, subtype‐specific prognostic and predictive value in adjuvant chemotherapy, and the distinct immune landscape of each subtype. Tumor‐derived Vimentin (VIM) was nearly expressed in triple‐negative SCLC tumors. VIM‐dominant SCLC subtype could independently predict ACT response and was associated with abundant but functionally impaired tumor‐infiltrating lymphocytes, which might help inform personalized immune therapeutic approaches.

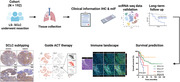

## 1 INTRODUCTION

Small cell lung cancer (SCLC) is an aggressive malignancy with early dissemination and dismal long‐term prognosis,^1^, ^2^ comprising about 15% of all newly diagnosed lung cancers.^3^ Despite having an initially robust response to platinum‐based chemotherapy, it rapidly acquired resistance and recurred eventually.^4^ While the addition of immunotherapy to front‐line chemotherapy shed light on a potential breakthrough of SCLC treatment, the survival benefits from this regimen were limited.^5^, ^6^ In contrast to the biomarker‐directed precision treatment strategies in non‐small cell lung cancer (NSCLC),^7^ the application of chemotherapy and immune checkpoint blockade (ICB) in unselected SCLC patients might underlie the unsatisfactory therapeutic effects.

Recently, a novel molecular subtype model of SCLC defined by four transcription factors (ASCL1, NEUROD1, POU2F3, and YAP1) was proposed^8^, ^9^ and these subtypes were reported to have distinct therapeutic vulnerabilities.^8^, ^10^, ^11^ Despite this, YAP1 failed to confirm a unique subtype in a subsequent validation study,^12^ and a novel, inflamed subtype (designated as SCLC‐I) was then proposed for those unclassified tumors.^13^ SCLC‐I tumors were considered mesenchymal with highly expressed mesenchymal markers such as Vimentin (VIM) and AXL and immune‐cell infiltrated. However, a suitable molecular marker for this putative SCLC‐I subtype remains importantly unknown.

Despite the potential feasibility of this new nomenclature and molecular subtyping method, no clinical surgical or front‐line therapy strategies have been changed to date. Few studies reported the prognostic value of SCLC subtypes concerning long‐term survival and adjuvant chemotherapy (ACT) response, possibly due to the relatively low number of clinical tumor samples and lack of outcome data. In addition, the immune landscape of each SCLC subtype and its association with ICB efficiency is crucial for clinical decision‐making. Gay et al. reported that SCLC‐I experienced the greatest survival benefit from atezolizumab immunotherapy based on the retrospective analysis of the tumor transcriptome data from the IMpower133 trial.^4^, ^13^ CD8^+^ T cell infiltration in tumor microenvironment was also found to be associated with pathological response to immune checkpoint blockade therapy in relapsed SCLC patients.^14^ These findings have prompted whether specific SCLC subtypes could have a practical application as an additional selection factor for immunotherapy.

Against this backdrop, we investigated suitable molecular markers for triple‐negative tumors on a large cohort of surgical resected SCLCs, evaluated its prognostic and predictive value for survival benefit in platinum‐based chemotherapy, and delineated the distinct immune landscape from multiplex immunofluorescence (mIF) and RNA sequencing data. VIM might be the novel marker for triple‐negative SCLC tumors with distinct prognostic value, ACT response, and immune landscape, which might be pivotal for future prospective trials designing subtype‐based precision therapy in SCLC.

## 2 RESULTS

### 2.1 Patients’ samples and characteristics

The detailed clinicopathologic features of enrolled 192 SCLC patients were summarized in Table 1. As expected, our cohort included more elders (*N* = 126, 65.6%), males (*N* = 164, 85.4%), and ever‐smokers (*N* = 147, 76.6%). Pathologically, most cases were stage I (40.6%). The presence rates of lymphovascular invasion (LVI) and visceral pleural invasion (VPI) were 50.0% and 77.6%, respectively. 73.7% of patients received postoperative adjuvant chemotherapy. The clinicopathological characteristics of patients performing subtype assessment (*N* = 145) are shown in Table S1. In total, 276 cores on TMAs containing SCLC tissues and paired nonadjacent uninvolved lung tissue and 16 slides were obtained from 145 surgical resected patients. There were at least two different cores per case on tissue microarrays (TMAs).

**TABLE 1:** Enrolled patient characteristics (*N* = 192).

Patient characteristics	SCLC	
	*N*	%
Age		
<60 years	66	34.4
≥60 years	126	65.6
Gender		
Female	28	14.6
Male	164	85.4
Smoking history		
Never	45	23.4
Ever	147	76.6
pTNM		
I	78	40.6
II	45	23.4
III	69	36.0
LVI		
Absent	96	50.0
Present	79	41.1
Unknown	17	8.9
VPI		
Absent	149	77.6
Present	32	16.7
Unknown	11	5.7
Histology type		
Pure	133	69.3
Combined	59	30.7
Adjuvant chemotherapy		
No	50	26.3
Yes	142	73.7

Abbreviations: LVI, lymphovascular invasion; SCLC, small cell lung cancer; VPI, visceral pleural invasion.

Unknown: Data were not available.

### 2.2 SCLC molecular subtypes defined by ASCL1, NEUROD1, POU2F3, and VIM

**FIGURE 1:**
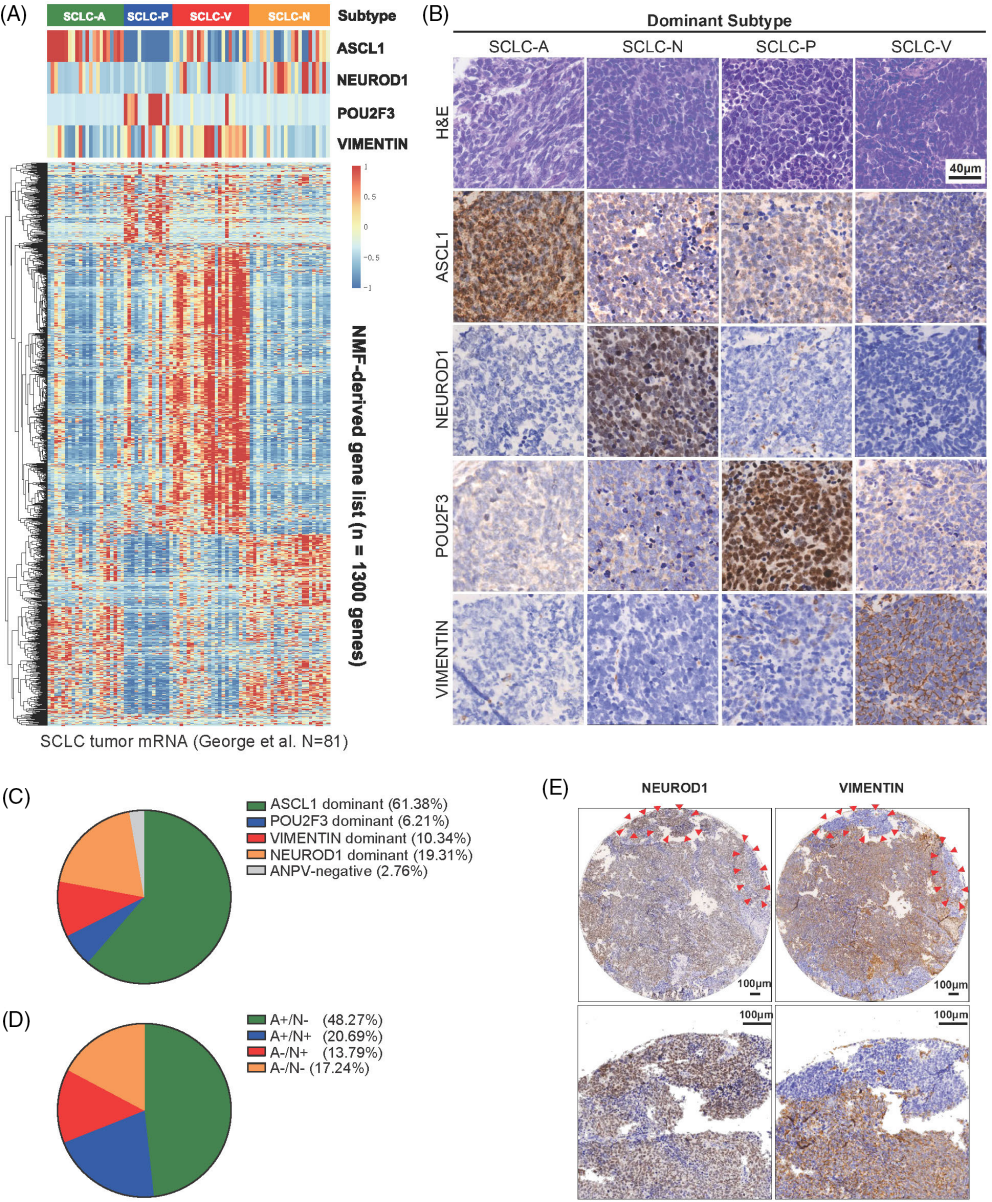
Small cell lung carcinoma (SCLC) **molecular subtypes defined by ASCL1, NEUROD1, POU2F3, and** Vimentin (VIM). (A) Differential expression of *ASCL1, NEUROD1, POU2F3*, and *VIM* across NMF‐defined 4 clusters using SCLC RNA sequencing data from George et al. (B) Representative images of immunohistochemistry (IHC) staining in primary SCLC tumors. (C) The proportion of each dominant molecular subtype tumor (*N* = 145). (D) The proportion of different co‐expression conditions for ASCL1 (A) and NEUROD1 (N). (E) A case with dual‐high NEUROD1 and VIM expression not only in the same cell populations but in spatially distinct subpopulations.

Previously, the transcription factor YAP1 failed to define a fourth molecular subtype of SCLC in both transcriptional and protein levels. The inflamed SCLC‐I subtype was subsequently proposed for these unclassified tumors. However, there was still no prevailing biomarker for this putative subtype, and mesenchymal markers such as VIM were reported to be the potential indicators. In order to verify this hypothesis, we first collected two transcriptome cohorts including surgical resected SCLC tumors for subtype exploration.^13^, ^15^ Four clusters were well separated by non‐negative matrix factorization (NMF) and identically high expression of VIM was observed in the fourth subtype (Figure 1A and Figure S1A). When compared with other classical mesenchymal markers such as AXL, N‐cadherin, FAP, and TWIST1, VIM performed the most outstandingly with the highest mRNA expression level in the fourth subtype (Figure S1B,C). Therefore, we chose VIM as the candidate and performed immunohistochemistry (IHC) on tumor TMAs. As shown in Figure 1B, four SCLC subtypes stained by ASCL1, NEUROD1, POU2F3, and VIM (SCLC‐A, N, P, and V) were successfully identified. The disperse distribution of these subsets yielded the proportions as follows: (1) SCLC‐A 61.38%; (2) SCLC‐N 19.31%; (3) SCLC‐V 10.34%; and (4) SCLC‐P 6.21% (Figure 1C). In our cohort, POU2F3 expression was exclusively restricted to other markers. Four cases (2.76%) were negative for all four markers (ANPV‐negative). The overall concordance rate of dominant subtype classification between different TMA cores was 96.1%, suggesting the high reproducibility of IHC assessment (Table S2). When evaluating the relationship between clinicopathological characteristics of SCLC tumors and different molecular subtypes, we found patient and tumor characteristics including gender, age, smoking history, pathological stages, histology type, and status of LVI and VPI showed no difference among SCLC molecular subtypes (Table S3).

Next, we explored the intra‐tumoral heterogeneity (ITH) and co‐expression conditions of molecular subtypes in SCLC tumors. Substantial co‐expression of ASCL1 and NEUROD1 either in spatially distinct or same cell populations has been demonstrated. In our study, the distribution of SCLC based on the presence of ASCL1 and NEUROD1 was as follows: (1) ASCL1‐only 48.27%, (2) NEUROD1‐only 13.79%; (3) ASCL1/NEUROD1 double‐positive 20.69%; and (4) ASCL1/NEUROD1 double‐negative 17.24% (Figure 1D). In one sample, notably, most areas of the cell population co‐expressed NEUROD1 and VIM, while two small clusters of tumor cells expressed NEUROD1 only (Figure 1E).

**FIGURE 2:**
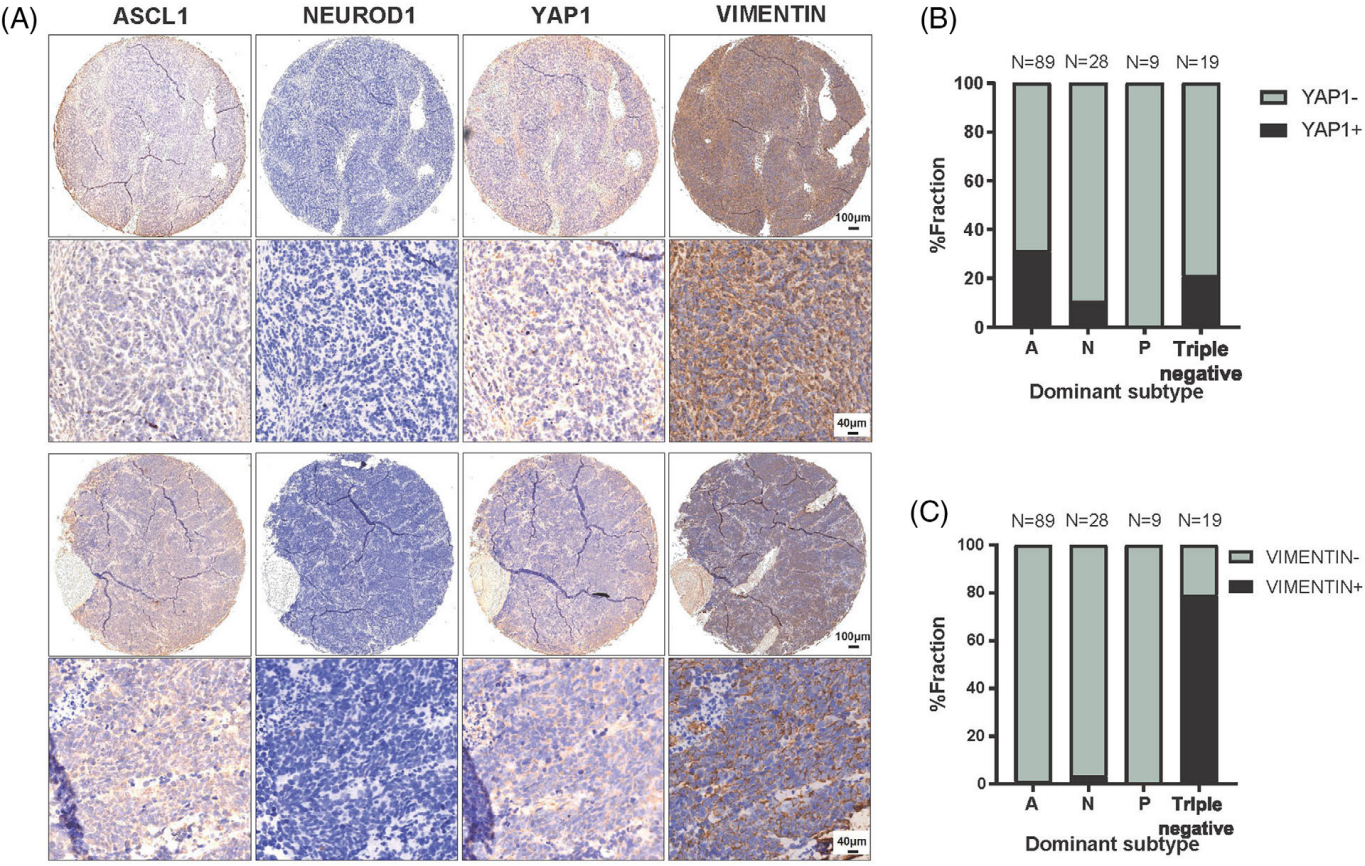
**Distribution of tumor‐derived YAP1 and** Vimentin (VIM) **expression in other dominant subtypes**. (A) Representative images of tumors with YAP1‐negative but VIM‐positive status. (B, C) The proportions of YAP1‐positive or VIM‐positive cases within small cell lung carcinoma (SCLC)‐A, SCLC‐N, SCLC‐P, and triple‐negative tumors.

Representative cases with YAP1‐negative but VIM‐positive were illustrated in Figure 2A. Consistent with previous reports, YAP1 expression was also distributed in other subtypes while VIM was nearly expressed restricted to triple‐negative samples (Figure 2B,C), which indicated VIM might be more suitable as the fourth subtype‐specific marker. Together these data reveal the feasibility of applying VIM as the marker of unclassified SCLC tumors. Subtype ITH and co‐expression at the single‐cell level existed substantially.

### 2.3 Predictive value and resistance mechanism of SCLC‐V subtype for ACT

**FIGURE 3:**
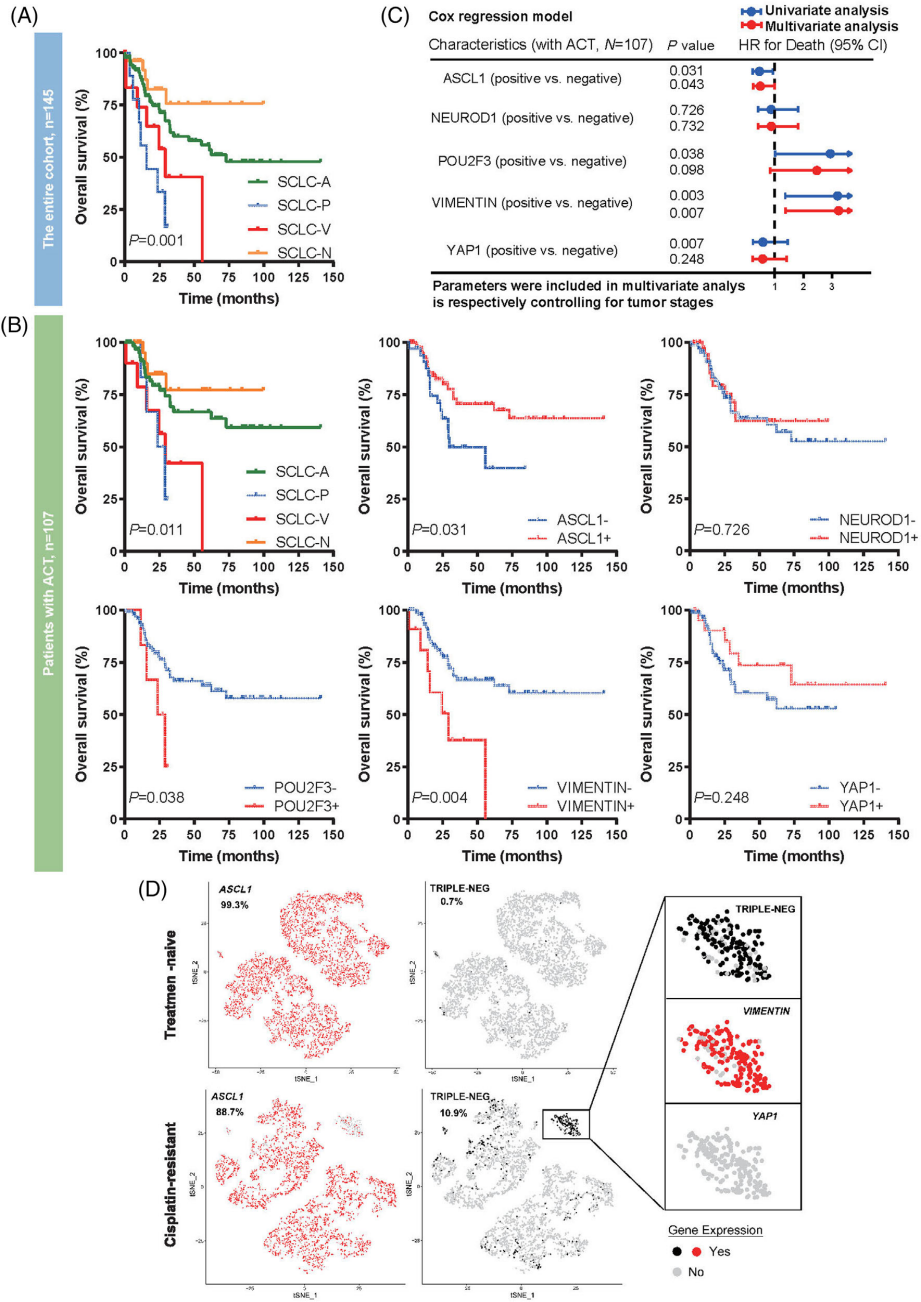
**Prognostic and predictive value of** small cell lung carcinoma (SCLC) **subtypes and the potential chemoresistance mechanism**. (A) The prognostic value of SCLC subtypes for overall survival in the entire cohort (*N* = 145). (B) The predictive value for chemotherapy benefit is stratified by the presence of each subtype‐defined marker in patients with chemotherapy (*N* = 107). (C) Multivariate cox analysis adjusting for tumor stages. (D) Analysis of single‐cell RNA sequencing data from treatment‐naive CDX models and paired cisplatin‐resistant models.

We next investigated the prognostic value of SCLC molecular subtypes stratified by ACT. The median follow‐up time was 55.8 months. In the entire cohort, this molecular subtype model had prognostic value for OS (*p* = 0.001, Figure 3A) and nearly significant for RFS (*p* = 0.073, Figure S2A). SCLC‐V and SCLC‐P subtype was associated with relatively poor prognosis. For the patient group that received adjuvant chemotherapy (Figure 3B and Figure S2B), differences in prognostic value were still significant for OS and nearly significant for RFS (*p* = 0.011 and *p* = 0.061, respectively).

The mechanism underlying chemoresistance in SCLC tumors was previously poorly understood and subtype‐specific susceptibilities to ACT were revealed until recently. Therefore, we evaluated the ACT benefit for survival based on the presence of each subtype marker (i.e., ASCL1, NEUROD1, POU2F3, VIM, and YAP1 as well). The results presented that in patients with ACT, there was a significant OS benefit in ASCL1‐positive tumors (*p* = 0.031) whereas patients with expression of POU2F3 or VIM were associated with poorer OS (*p* = 0.038 and *p* = 0.004, respectively; Figure 3B). Further multivariate analysis controlling for tumor pathological stages confirmed the independent predictive value of ASCL1 and VIM but not POU2F3 for ACT benefit (Figure 3C). Multivariate analysis of RFS is also shown in Figure S2C.

To further validate the distinct vulnerabilities to chemotherapy of SCLC‐A and SCLC‐V, we used public single‐cell RNA sequencing data from two CDX models (MDA‐SC53 and MDA‐SC68, merged as one sample), containing treatment‐naive and paired cisplatin‐resistant tumors after relapse. When compared with the treatment‐naive sample, a modest decrease of ASCL1‐positive cell proportion along with the emergence of a triple‐negative cluster was observed in the cisplatin‐resistant sample, and this cluster featured a high VIM but no YAP1 expression (Figure 3D).

Together, these observations suggest the potential prognostic and predictive value of SCLC molecular subtypes, and demonstrate contrary platinum vulnerabilities of SCLC‐A and SCLC‐V in clinical patient cohort for the first time.

### 2.4 Distinct immune contexture in SCLC‐V subtype

**FIGURE 4:**
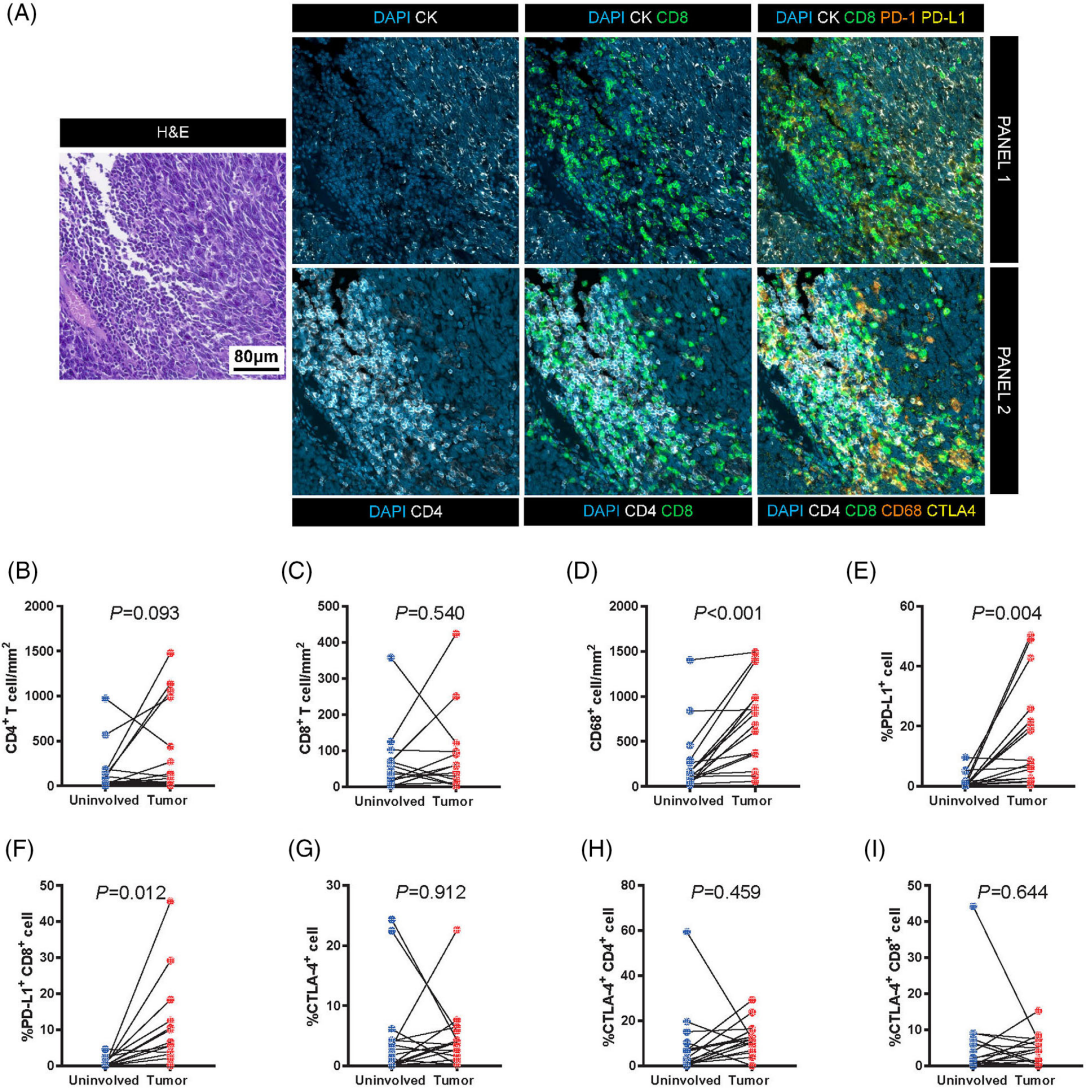
**The immune contexture differed between** small cell lung carcinoma (SCLC) **tumors and paired normal adjacent tissues**. (A) Representative images of multispectral immuno‐fluorescence (mIF) staining by two panels. (B–J) Quantification of counts of CD4^+^ T‐cells, CD8^+^ T‐cells, CD68^+^ cells and proportions of PD‐L1^+^ cells, PD‐L1^+^CD8^+^ cells, CTLA4^+^ cells, CTLA4^+^CD4^+^ cells, and CTLA4^+^CD8^+^ cells in tumors and paired normal lung tissues (*N* = 16).

To investigate the specific immune contexture in primary SCLC tumors, we first performed mIF on 16 pairs of tumors and normal adjacent tissues (Figure 4A). Quantification using mIF demonstrated that CD68^+^ cell counts (Figure 4D) were relatively higher in tumors when compared to adjacent uninvolved lung tissues (*p* < 0.001). In addition, the percentages of PD‐L1^+^ cells within the total cells (Figure 4E) and PD‐L1^+^CD8^+^ T‐cells (Figure 4F) within the total CD8^+^ immune cells were significantly higher in tumors compared to adjacent tissues (*p* = 0.004 and *p* = 0.012, respectively), which was not observed in other cell types (Figure 4B,C,G–I). PD‐1 expression was not detected in most cases and only three samples contained at least 1% PD‐1‐stained cells within CD8^+^ immune cells, which was therefore not included in subsequent analyses. When restricted our analysis to patients stratified by pathological stages, the immune cell infiltration levels and expression of immune checkpoints including PD‐L1 and CTLA4 were basically higher in stage I compared with stage II‐III tumors (Figure S3).

**FIGURE 5:**
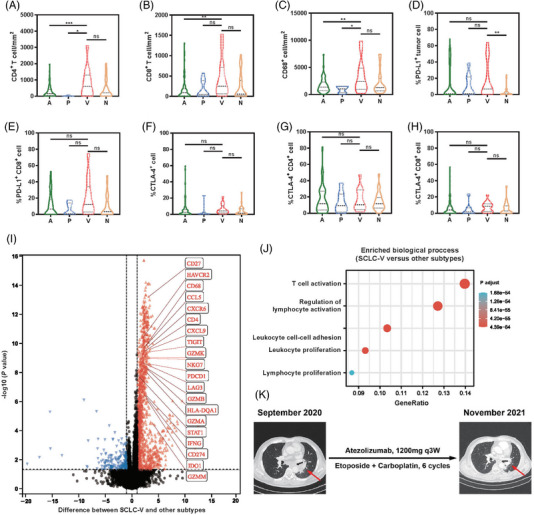
**The association between** small cell lung carcinoma (SCLC) **molecular subtypes and tumor immune landscape**. (A–C) The distribution of immune cell infiltration in different SCLC subtypes. (D‐H) Subtype‐specific expression levels of PD‐L1 and CTLA4 on immune cells. (I) Volcano plot for normalized log2‐transformed differential gene expression between SCLC‐V and other subtypes. (J) Enrichment biological process analysis for genes with mRNA increase by twofold or greater between SCLC‐V and other subtypes. (K) A case with Vimentin (VIM)‐dominant SCLC received anti‐PD‐L1 therapy after tumor relapse.

Next, we evaluated the association between SCLC molecular subtypes and tumor immune microenvironment. As shown in Figure 5A–C, CD4^+^ T‐cell, CD8^+^ cytotoxic T‐cell and CD68^+^ macrophage cell counts were relatively higher in the SCLC‐V subtype compared with other subtypes, suggesting a greater abundance of immune cell infiltration. SCLC‐V subtype was also associated with the highest median of the PD‐L1^+^ cell, PD‐L1^+^CD8^+^ T‐cell, and CTLA4^+^CD8^+^ T‐cell proportions (Figure 5D,E,H). Despite the difference was not statistically significant in mIF analysis (Figure 5D,E,H), the mRNA expression of CD8A and PD‐L1 in the SCLC‐V subtype was significantly higher than other subtypes (*p* = 0.044 and *p* < 0.001, respectively. Figure S4). When separated patients into high/median/low‐expression group of PD‐L1^+^CD8^+^T cell or CTLA4^+^CD8^+^T cell based on the combination of PD‐L1/CD8A or CTLA4/CD8A mRNA expression level (e.g., high expression of both PD‐L1 and CD8A represented as “high” group), such two immunosuppressive cells were also more abundant in SCLC‐V subtype (Figure S4E), which was concordant with the fluorescence results. However, we did not observe any subtype‐specific distribution concerning the percentages of CTLA4^+^ cell and CTLA4^+^CD4^+^ cell (Figure 5F,G). We conducted further validation in the George et al. cohort analyzing mRNA expression fold changes in SCLC‐V subtype compared with other subtypes. Increased immune‐related markers were also confirmed in SCLC‐V subtype, including cytotoxic function mediated genes such as GMZA, GMZB, and IFNG; immune cell inflamed genes such as CD4, CD68, and CXCL9; and immune checkpoint molecules such as CD274, TIGIT and LAG3 (Figure 5I). Further analysis of genes with mRNA increasing by two folds or greater revealed that the top activated biological processes in SCLC‐V subtype included lymphocyte activation and proliferation (Figure 5J).

Tumors with inflamed tumor microenvironments have been demonstrated to be more likely to respond to ICB therapy. Therefore, we retrieved the patients receiving anti‐PD‐L1 immunotherapy and identified one patient with VIM‐dominant SCLC. This patient, a male, 62 years old, and a former smoker, underwent left upper lobectomy and mediastinal lymphadenectomy in June 2019, and he was diagnosed with disease relapse in the left lung and pleura. He began treatment with 1200 mg of atezolizumab every 3 weeks combined with six cycles of etoposide and carboplatin in September 2020 and showed a durable partial response for 14 months, with no evidence of progression or active disease at the latest follow‐up in November 2021 (Figure 5K). These findings suggested that VIM‐dominant SCLC might benefit from ICB therapy, despite the aggressive tumor biological behavior and unfavorable predictive value for adjuvant chemotherapy treatment.

### 2.5 Tumor immunophenotypic signatures with clinical impact

**FIGURE 6:**
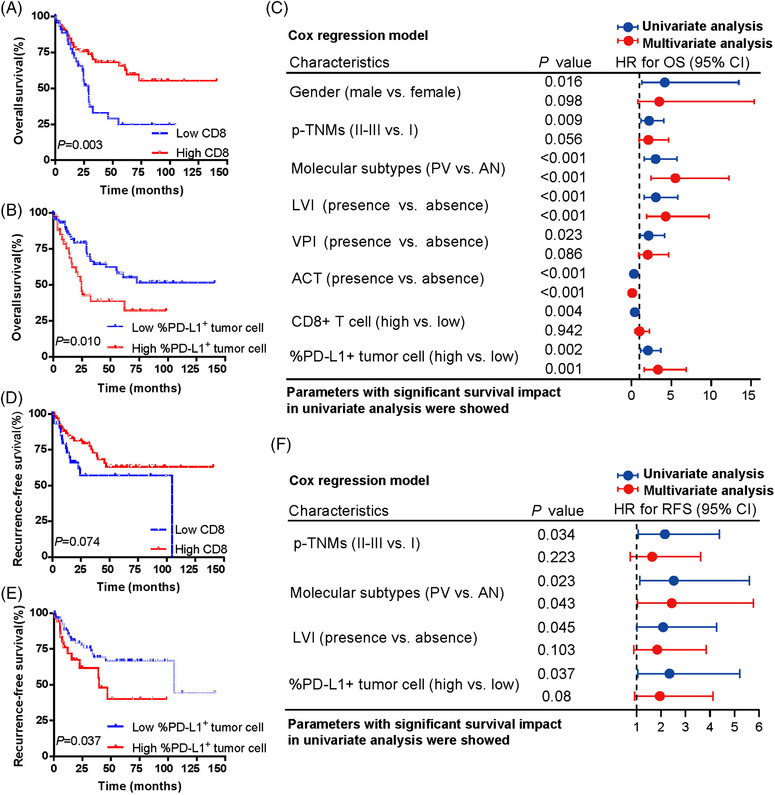
**The prognostic value of immunophenotypic signature in primary** small cell lung carcinoma (SCLC). (A–C) The prognostic value of CD8^+^T cells and PD‐L1^+^ tumor cells for overall survival and the corresponding multivariate Cox regression analysis. (D–F) The prognostic value of CD8^+^T cells and PD‐L1^+^ tumor cells for recurrence‐free survival and the corresponding multivariate Cox regression analysis.

To further investigate the prognostic value of each immunophenotypic signature in primary SCLC, we conduct survival analysis based on the corresponding cutoff value (Table S4). As shown in Figure 6, there was a significant OS benefit in patients with high CD8^+^ T‐cell (*p* = 0.003, Figure 6A), with a nonsignificant trend toward a RFS benefit (*p* = 0.074; Figure 6D). Moreover, patients with a lower proportion of PD‐L1^+^ tumor cells within all tumor cells experienced a significantly longer OS (*p* = 0.010, Figure 6B) as well as RFS (*p* = 0.037, Figure 6E) than those with a higher proportion of PD‐L1^+^ tumor cells. The prognostic value of other immune markers was showed in Figure S5.

Considering that stage is a major factor of OS and RFS in SCLC, multivariate analyses were further carried out for immune cell infiltration and other potential clinical prognostic variables (Figure 6C,F). SCLC molecular subtypes (SCLC‐P/V vs. SCLC‐A/N) had a statistically significant impact on both OS (*p* < 0.001) and RFS (*p* = 0.043) in multivariate model. A higher proportion of PD‐L1^+^ tumor cell infiltration was the only immune‐related marker that was associated with significantly worse OS (*p* < 0.001) in multivariate analysis. Patients with positive LVI status had a statistically significant shorter OS (*p* < 0.001). Collectively, our results indicated that immunophenotypic signatures as well as molecular subtypes were independently prognostic factors of recurrence and death in primary SCLC tumors.

## 3 DISCUSSION

Clinical therapeutic research in SCLC has entered the fast track in recent years, underpinning this progress has been the proposal of a new model of SCLC subtypes defined by relative expressions of ASCL1, NEUROD1 with a high NE program, and POU2F3, YAP1 with a non‐NE program, named as SCLC‐A, SCLC‐N, SCLC‐P, and SCLC‐Y, respectively.^9^ Subsequent two studies failed to identify a distinct SCLC‐Y population^12^, ^16^ and a unique SCLC‐I subtype with epithelia‐mesenchymal transition (EMT) and inflamed phenotype was newly proposed.^13^ However, a dominant lineage marker in this SCLC‐I subtype has still not been well identified. In the current study, using TMAs and specimen slides from a relatively large cohort (*N* = 192) of primary resected SCLC, we assessed the tumor‐derived protein expressions of ASCL1, NEUROD1, POU2F3, YAP1 as well as VIM, a mesenchymal marker which may be a potential assay to define SCLC‐I. Our findings confirmed that SCLC‐A (61.38%) counted for the majority of SCLC, followed by SCLC‐N (19.31%) and SCLC‐P (6.21%) subtypes. A minor subset of SCLC (*N* = 19) lacked all of these transcriptional regulators (triple‐negative) while only two of these cases expressed low level of YAP1. Conversely, fifteen cases among triple‐negative SCLC tumors expressed VIM with a relatively strong pattern, which was confirmed in transcriptional data from two public cohorts using NMF clusters.^15^, ^17^ The above evidences indicated VIM was the prevailing predictive biomarker compared with YAP1 in these cohorts. Although VIM is well known as an effector molecule rather than a dominant transcriptional regulator, such a finding is still of significant clinical implications, as it offers more convenient and accurate IHC‐based criteria for pathologists to classify most SCLC tumors into one of the four subtypes, and consequently guides targeting and personalized therapy strategies. Not like POU2F3, notably, VIM expression was not strictly mutually exclusive of other markers. The aforementioned unique case exhibited dual‐high NEUROD1 and VIM expression not only in same cell populations but in spatially distinct subpopulations, which indicate the subtype transition or plasticity within a single tumor.

Is subtype assignment in de novo disease associated with long‐term survival, ACT response, and immune milieu? Although substantial data have been reported regarding SCLC subtypes and related clinicopathological features at transcriptional and protein levels,^12^, ^13^, ^18^ there is currently very limited information on the prognostic and predictive value of this model, mainly due to the highly aggressive behavior and rare clinical samples of SCLC. In the George et al. cohort, there were no significant differences in RFS or OS regarding different subtypes based on 81 resected SCLC patients.^13^ With an adequate follow‐up time (median 55.8 months), we revealed that the long‐term survival of patients with non‐NE subtypes (SCLC‐P and SCLC‐V) was poorer than NE subtypes (SCLC‐A and SCLC‐N), which was concordant with the results from Delphine et al. separating patients into NE and non‐NE subgroups.^19^ SCLC subtyping remained as an independent prognosticator even after adjusting clinicopathological confounders. These findings can help clarify the prognostic role of SCLC subtyping model in resected SCLC, which may benefit future tumor staging system and stratification of high‐risk groups.

Two hypotheses account for the distinct prognostic role of each SCLC subtype. The first suggests the innate biological heterogeneity of different tumor subtypes, including proliferation rate, replication stress, and the ability to cope with microenvironment stresses.^20^ However, both our and previous studies demonstrated equivalent distribution of Ki‐67 proliferation indices and pathological stages in SCLC subtypes.^12^ Another reasonable hypothesis is that distinct platinum‐specific vulnerabilities exist between subtypes. Not like NSCLC wherein adjuvant chemotherapy is mainly determined by tumor stage and pathological subtypes,^21^ platinum‐based chemotherapy is still a “one‐size fits all” regimen for both limited and advanced SCLC. Subtype‐specific vulnerabilities to cisplatin have been preliminarily reported in vitro and the increase of chemo‐resistant cell populations is considered as the underlying mechanism.^13^ In this study, therefore, we evaluated the ACT benefit based on the presence of each marker instead of the dominant subtype. The results revealed that patients with ASCL1‐positive SCLC benefit the most while patients with VIM‐positive tumors did the opposite. Further analysis of scRNAseq data from two CDX models confirmed the emergence of VIM‐featured cells after cisplatin‐resistance. To date, no clear association between SCLC subtype and chemotherapy resistance has been reproducibly found. Recent data suggest a role for transdifferentiation to a non‐NE subtype (such as SCLC‐I) as a potential mechanism of chemoresistance.^22^ A low frequency of ASCL‐1 positivity was found in relapsed tumors after chemotherapy, potentially through suppressing Wnt and Notch signaling pathways, the latter of which could lead to a switch from NE to non‐NE cell phenotype.^23^ The above findings may promote personalized therapy for patients with acquired chemoresistance and postoperative recurrence. Nevertheless, these analyses were retrospective, and clinical prospective validations are still warranted.

Another important contribution of this work is the comprehensive assessment of subtype‐specific differences in tumor immune milieu. A previous study has reported an inflamed microenvironment in the SCLC‐I subtype with greater infiltration of several immune cell populations and higher expression of countless immune checkpoint molecules based on transcriptional profiling.^13^ Remarkably, our study confirmed at the protein level that CD4^+^ T‐cell, CD8^+^ cytotoxic T‐cell, and CD68^+^ macrophage cells were abundant but functionally impaired in the VIM‐defined SCLC subtype (SCLC‐V). The proportions of PD‐L1^+^ cell, PD‐L1^+^CD8^+^ T‐cell, and CTLA4^+^CD8^+^ T‐cell were relatively higher in the SCLC‐V subtype compared with other subtypes. This from a side, confirmed the feasibility of applying VIM as the marker of these triple‐negative SCLC tumors. Expression of VIM on tumor cells with partial or complete loss of epithelial marker expression such as EpCAM represents hybrid or full EMT status. EMT has classically been regarded as a tumor development phenotype as well as a tumor immune evasion marker. Overexpression of EMT transcription factors such as Prrx1 could significantly increase tumor invasiveness and subsequently promote distant metastasis.^24^ Several studies have also shown that EMT is associated with immunosuppressive cell number and the expression of immune checkpoints such as PD‐L1.^25^, ^26^ Molecularly, Snail, and Zeb1 serve as EMT transcriptional factors and produce chemokines to attract immunosuppressive cells or promote the expression of immunosuppressive checkpoint molecules,^27^, ^28^ which may lead to the tumor immunosuppressive microenvironment in SCLC‐V subtype. Further survival analysis revealed that a high proportion of PD‐L1^+^ cells remained an independent poor prognosticator after adjusting for clinicopathologic confounders. Tumor infiltrating lymphocytes with high co‐inhibitory receptor expression such as CTLA4, PD‐1, and LAG3 have been regarded as functionally exhausted rather than activated.^29^, ^30^ Moreover, PD‐L1 expression not only on tumor cells but also on immune cells can lead to the inhibition of effector T‐cell activity.^31^, ^32^, ^33^, ^34^ In the Keynote‐028 and Keynote‐158 studies,^35^, ^36^ combined PD‐L1 expression on tumor and immune cells could better predict response to ICB. Therefore, such an immune microenvironment may provide new insights into the predictive biomarker exploration for ICB targeting PD‐L1 with or without concurrent targeting of CTLA4.

Our study has several limitations. Caution should be taken when applying our findings to extensive‐stage SCLCs since all the tumors included in the current study are local‐staged. Second, despite including a relatively large cohort of primary SCLCs, the sample numbers of SCLC‐P/V subtypes are still small, and further larger cohort studies are warranted. Lastly, the retrospective nature of the study raised the risk of selection and recall bias in patient and sample selection. In summary, our study confirmed and modified the subtype‐based SCLC classification by introducing VIM to be the novel marker for those triple‐negative tumors in primary SCLC. With complete clinicopathologic and survival information, we confirmed the independent prognostic value and distinct predictive roles of SCLC subtypes for frontline chemotherapy in the real‐world cohort. This work also delineates the distinct immune landscape of the SCLC‐V subtype, which is important for future prospective trials designing of subtype‐based patient selection strategy to help optimize the use of immunotherapy in SCLC.

## 4 MATERIALS AND METHODS

### 4.1 Study population

This study was registered in the Chinese Clinical Trial Registry (ChiCTR2200061584) and approved by the institutional review board (Fudan University Shanghai Cancer Center, IRB 2008223−9). From November 2006 to April 2021, we consecutively collected 192 SCLC patients who underwent complete surgical resection with complete clinical characteristic data. All cases were reviewed by a single pathologist to confirm the original diagnosis of SCLC and sufficient tumor samples of at least 20% enrichment in the resection samples. After an independent review of all hematoxylin and eosin (HE) slides and assessment of formalin‐fixed paraffin‐embedded (FFPE) block quality, 129 cases were constructed into tumor tissue microarray (TMA) and 16 cases were analyzed in slides. These 145 patients with available tumor specimens subsequently performed IHC to assess SCLC subtypes and mIF to assess the tumor immune microenvironment (Figure S6). Data on clinicopathological variables were obtained by reviewing patient medical records specifically for this study purpose. Recurrence‐free survival (RFS) and overall survival (OS) were recorded based on follow‐up clinic or telephone. Another SCLC patient cohort was from George et al. with publicly available tumor gene expression data via the European Genome‐phenome Archive under the accession code EGAS00001000925.^17^ Informed consents were waived because it was a retrospective study.

### 4.2 TMA construction

SCLC TMA was constructed in the Department of Pathology, Fudan University Shanghai Cancer Center using the automated TMA Grand Master (3DHISTECH) and TMA Control software. The HE slides were reviewed to select the most viable tumor and uninvolved lung tissue areas. Finally, 260 cores (two or three crores per case) were punched in corresponding tumor areas, and an additional 16 crores (one crore per case) in lung tissue areas, with 2 mm in diameter.

### 4.3 Immunohistochemistry

TMAs and slides were stained for subtype‐defined markers of SCLC including ASCL1 (clone 24B72D11.1, dilution 1:100; BD Biosciences), NEUROD1 (clone EPR17084, dilution 1:50; Abcam), POU2F3 (clone 6D1, dilution 1:100; Santa Cruz), YAP1 (clone 63.7, dilution 1:2000; Santa Cruz) and Vimentin (clone D21H3, dilution 1:100; Cell Signaling Technologies) using Anti‐mouse/rabbit IHC Detection Kit (PK10006; Proteintech) according the manufacturer's protocol. Two independent pathologists reviewed the stained slides in a blinded fashion. The expression of each marker from tumor cells was assessed by histoscore (H‐score, range 0–300), which was calculated by multiplying the proportion of positive tumor cells (0%–100%) by the intensity of positive staining (no staining = 0, weak staining = 1, moderate staining = 2, and strong staining = 3).^37^, ^38^ A dominant marker is defined as the marker with the highest H‐score. In SCLC with combined SCLC and NSCLC components, IHC scores reflect expression exclusively in the SCLC component.

### 4.4 Multiplex IF

mIF analysis was performed on TMAs as previously described. Briefly, TMA sections were stained using antibodies against cytokeratins (clone C‐11, dilution 1:250; Abcam), PD‐1 (clone NAT105, dilution 1:100; Abcam), PD‐L1 (clone E1L3N, dilution 1:100; Cell Signaling Technologies), CD8 (clone D8A8Y, dilution 1:200; Cell Signaling Technologies), CD4 (clone EP204, dilution 1:100; Cell Signaling Technologies), CD68 (clone D4B9C, dilution 1:400; Cell Signaling Technologies) and CTLA4 (clone CAL49, dilution 1:200; Abcam). All antibodies were linked with one of the fluorophores from the Opal 7 IHC kit, (NEL797001KT; Akoya Biosciences). Sections were scanned by a tissue imaging system (Aperio Versa 8; Leica). Imaging analysis was performed with quantitative image analysis software (Halo; Indica Labs).

### 4.5 Statistical analysis

The scRNAseq data from two SCLC circulating tumor cells (CTC)‐derived xenograft (CDX) models developed from treatment‐naive patients and paired with two platinum‐relapsed models (“cis‐relapsed”) after cisplatin treatment were selected for downstream analyses. Raw scRNAseq data is available in the GEO Database under the accession code: GSE138474.^39^ Briefly, the “treatment‐naive” and “cis‐relapsed” samples were first pooled together respectively. Cells that have less than 3,000 expressed genes and genes that were expressed in less than 10% of cells were filtered out. Normalization, adjustment of cell cycle effects, and principle component analysis were performed using the “NormalizeData()”, “ScaleData()” and “RunPCA()” functions of the SEURAT package v4.1.0.^40^ Cell populations were identified using the “FindClusters()” function with resolution set to 0.6.

NMF^41^ was applied to the RNA‐seq data from George et al.^17^ and Sato et al.^15^ using a previously reported gene list.^13^ Maximization of cophenetic correlation values was used to guide the optimal number of distinct clusters and finally the four‐cluster option was selected. The expression of each subtype‐defined marker of SCLC was presented stratified by clusters. Correlations between two categorical variables were analyzed using the Chi‐square test or Fisher's exact test. Student's t‐test was used to compare continuous variables. The RFS and OS were investigated by log‐rank test to compare differences between groups. Cox proportional hazard regressions were used to predict recurrence and death status. Variables with a *p‐*value less than 0.05 in univariate analysis were entered into multivariate survival analysis.

The statistical analysis was conducted in R Statistical Language (version 4.1.2). All tests were two‐tailed. Statistical significance was set at *P* < .05. This work has been reported in line with the STROCSS criteria.^42^

## AUTHOR CONTRIBUTIONS

All authors studied the concept and design; All authors performed acquisition, analysis, or interpretation of data; All authors drafted the manuscript; All authors performed critical revision of the manuscript for important intellectual content. C.D., Y.W., F.F., and D.L. did the statistical analysis; H.C., Y.Z., and C.D. obtained funding; Y.Z., H.C., and Y.L. provided administrative, technical, or material support; Y.Z., H.C., Y.L. did the study supervision; all authors provided final approval of the version to be published; All authors agreed to be accountable for all aspects of the work in ensuring that questions related to the accuracy or integrity of any part of the work are appropriately investigated and resolved.

## CONFLICT OF INTEREST STATEMENT

The authors declare no conflict of interest.

## ETHICS STATEMENT

This study was registered in the Chinese Clinical Trial Registry (ChiCTR2200061584) and approved by the institutional review board (Fudan University Shanghai Cancer Center, IRB 2008223−9).

## CONSENT TO PARTICIPATE

Informed consent was waived because it was a retrospective study.

## Supporting information

Supporting Information

## Data Availability

The datasets used and analyzed during the current study are available from the corresponding author upon reasonable request.
