# Supplementary material for: Tumor‐derived Vimentin as a novel biomarker for distinct subtypes predicting adjuvant chemotherapy resistance and T‐cell‐inflamed phenotype in small cell lung cancer
Source: MedComm (2020). 2023 Oct 1;4(5):e370. doi: 10.1002/mco2.370 (PMC10542987; doi:10.1002/mco2.370)
Supplement: Supplementary file 1 — Supporting Information [file MCO2-4-e370-s001.pdf]

Supplementary Materials for:

Tumor-derived Vimentin as a novel biomarker for distinct subtype predicting adjuvant chemotherapy resistance and T-cell-inflamed phenotype in small cell lung cancer

Chaoqiang Deng, MD[*1,2,3], Yue Wang, MD[*2,3,4], Fangqiu Fu, MD[*1,2,3], Di Li, MD[1,2,3], Qiang Zheng, MD[2,3,4], Yan Jin, MD[2,3,4], Yuan Li, MD, PhD[#2,3,4], Haiquan Chen, MD, PhD[#1,2,3] and Yang Zhang, MD[#1,2,3]

Authors' Affiliations:

[1]Department of Thoracic Surgery and State Key Laboratory of Genetic Engineering, Fudan University Shanghai Cancer Center, Shanghai, China;

[2]Institute of Thoracic Oncology, Fudan University, Shanghai, China;

[3]Department of Oncology, Shanghai Medical College, Fudan University, Shanghai, China;

[4]Department of Pathology, Fudan University Shanghai Cancer Center, Shanghai, China;

[#]Corresponding Author:

[#]Dr. Yang Zhang, E-mail: fduzhangyang1987@hotmail.com;

[#]Dr. Haiquan Chen, E-mail: hqchen1@yahoo.com;

[#]Dr. Yuan Li, E-mail: lumoxuan2009@163.com;

[*]These authors equally contributed to this work.

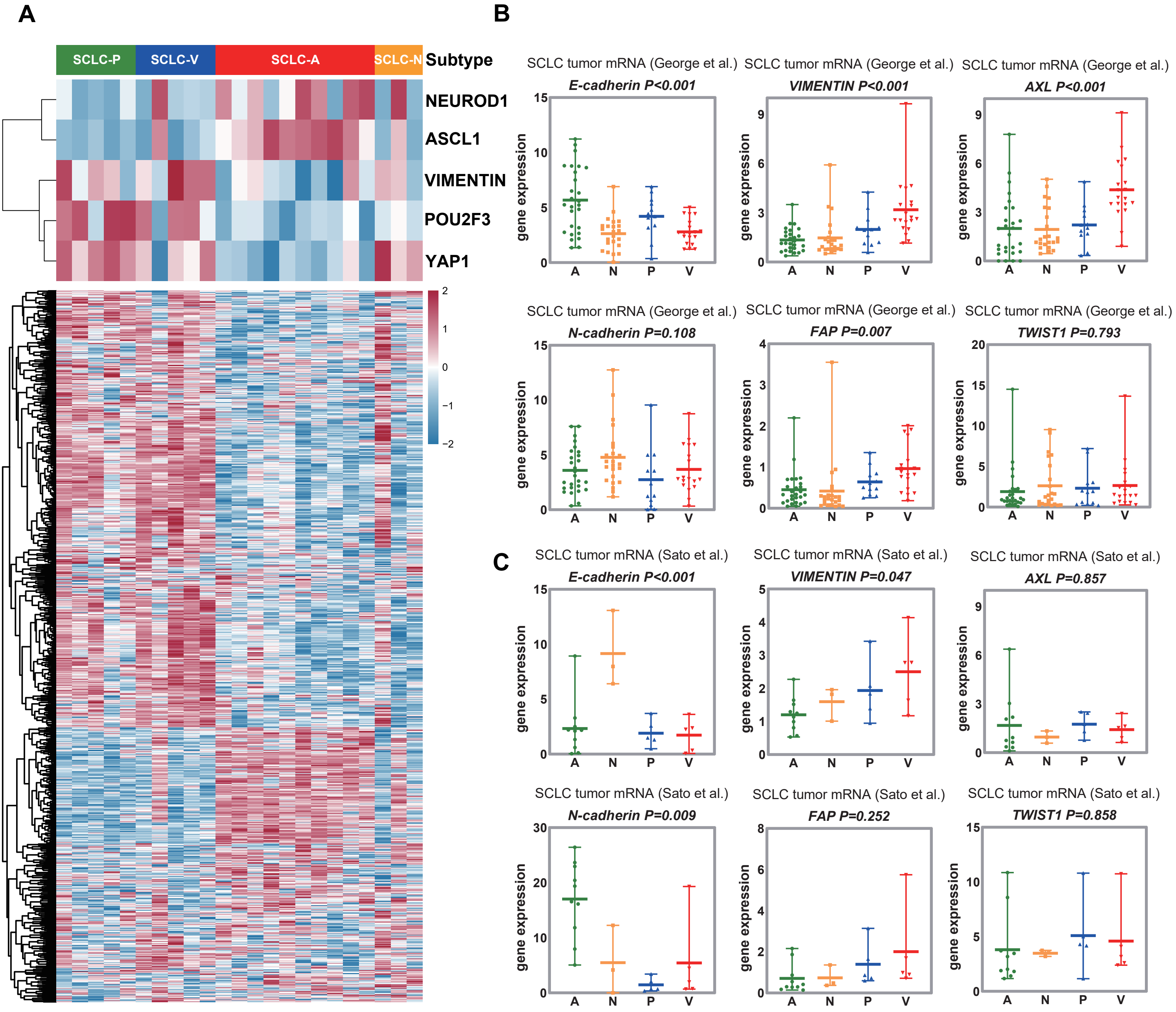

Fig. S1 SCLC molecular subtypes defined by ASCL1, NEUROD1, POU2F3 and VIM. (A) Differential expression of potential biomarkers using RNA sequencing data from Sato et al. **(B-C)** Expression of classical EMT markers in each SCLC subtype.

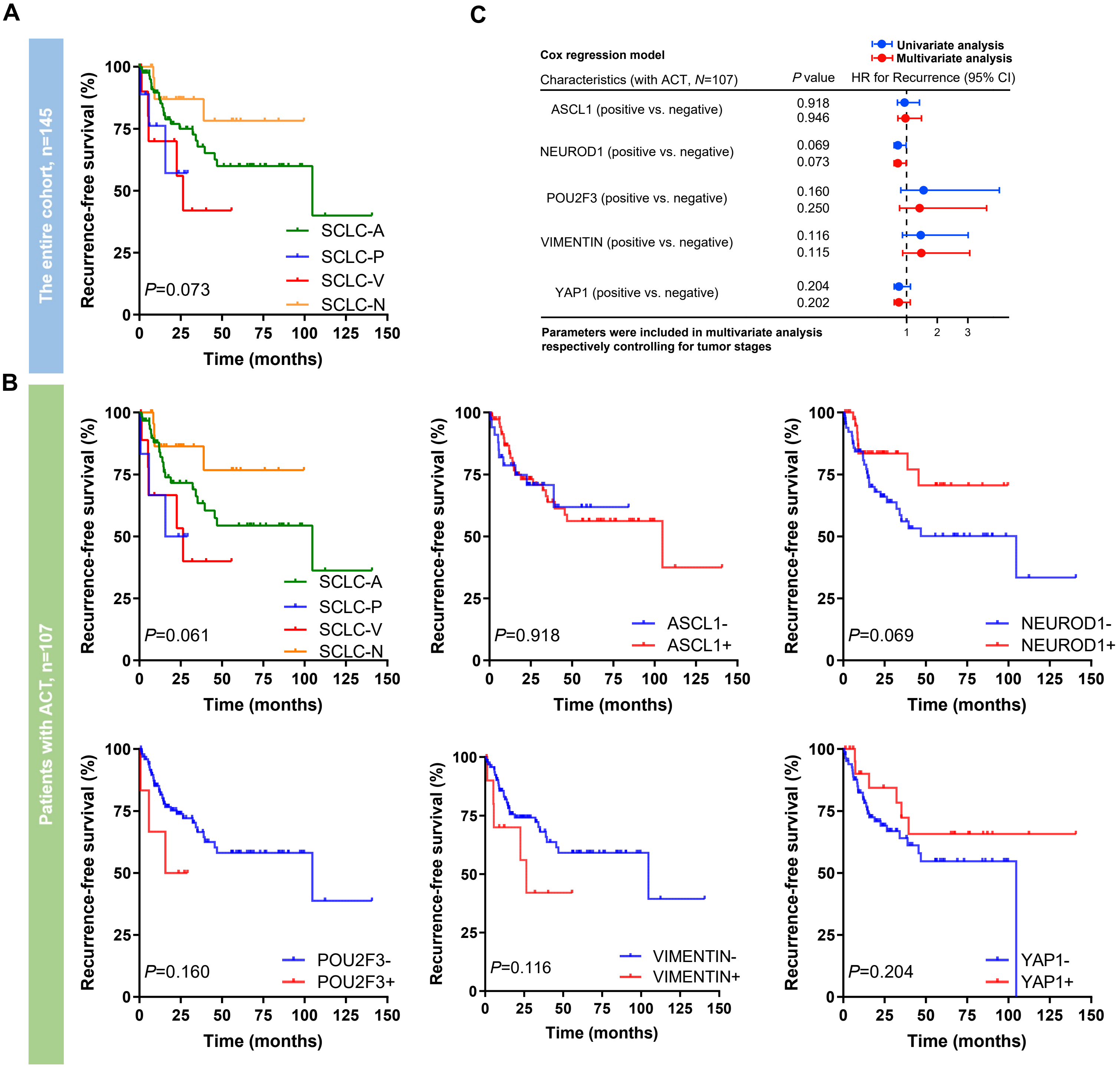

Fig. S2 Prognostic and predictive value of SCLC subtypes regarding recurrence-free survival. (A) The prognostic value of SCLC subtypes for recurrence-free survival in the entire cohort (N=129). **(B)** The predictive value for chemotherapy benefit stratified by the presence of each subtype-defined marker in patients with chemotherapy (N=97). **(C)** Multivariate cox analysis adjusting for tumor stages.

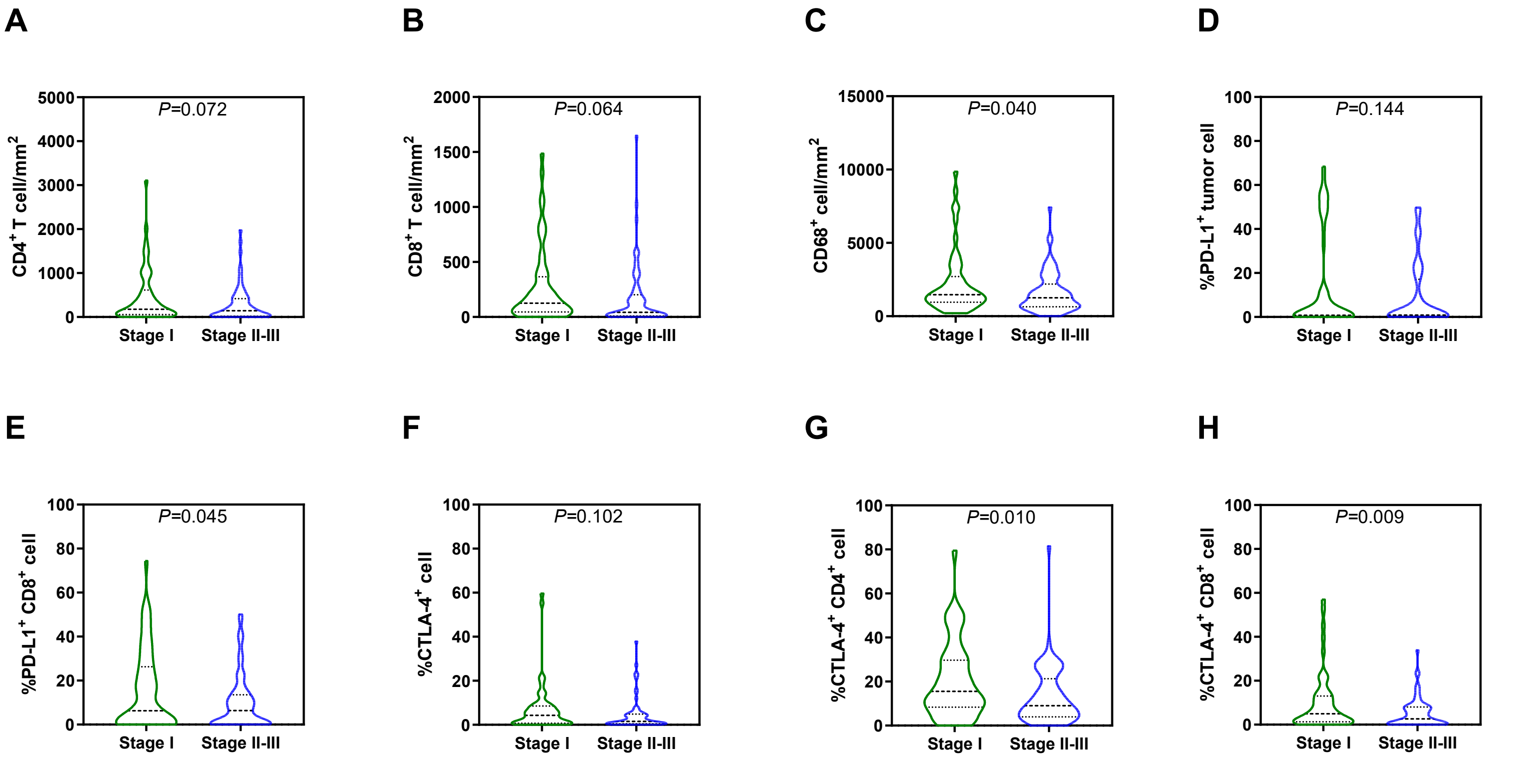

Fig. S3 Stratification analysis of immune cell infiltration and checkpoints expression by pathological stages.

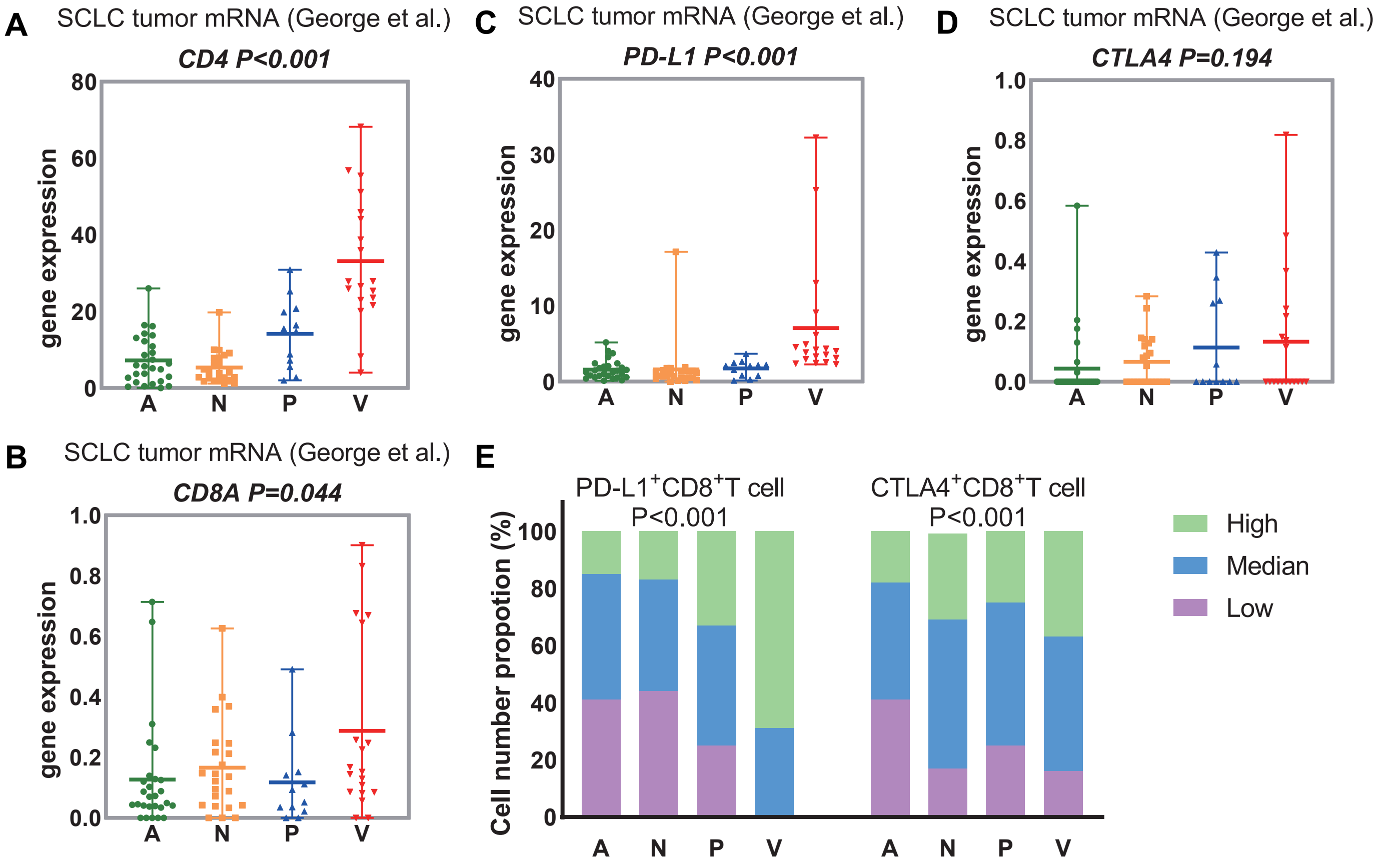

Fig. S4 The association between SCLC molecular subtypes and tumor immune biomarkers using RNA-seq data. (A-D) Different expression level of CD4, CD8A, PD-L1 and CTLA4 in each SCLC subtypes. **(E)** Different proportion of PD-L1^{+}CD8^{+}T cell and CTLA4^{+}CD8^{+}T cell in SCLC subtypes.

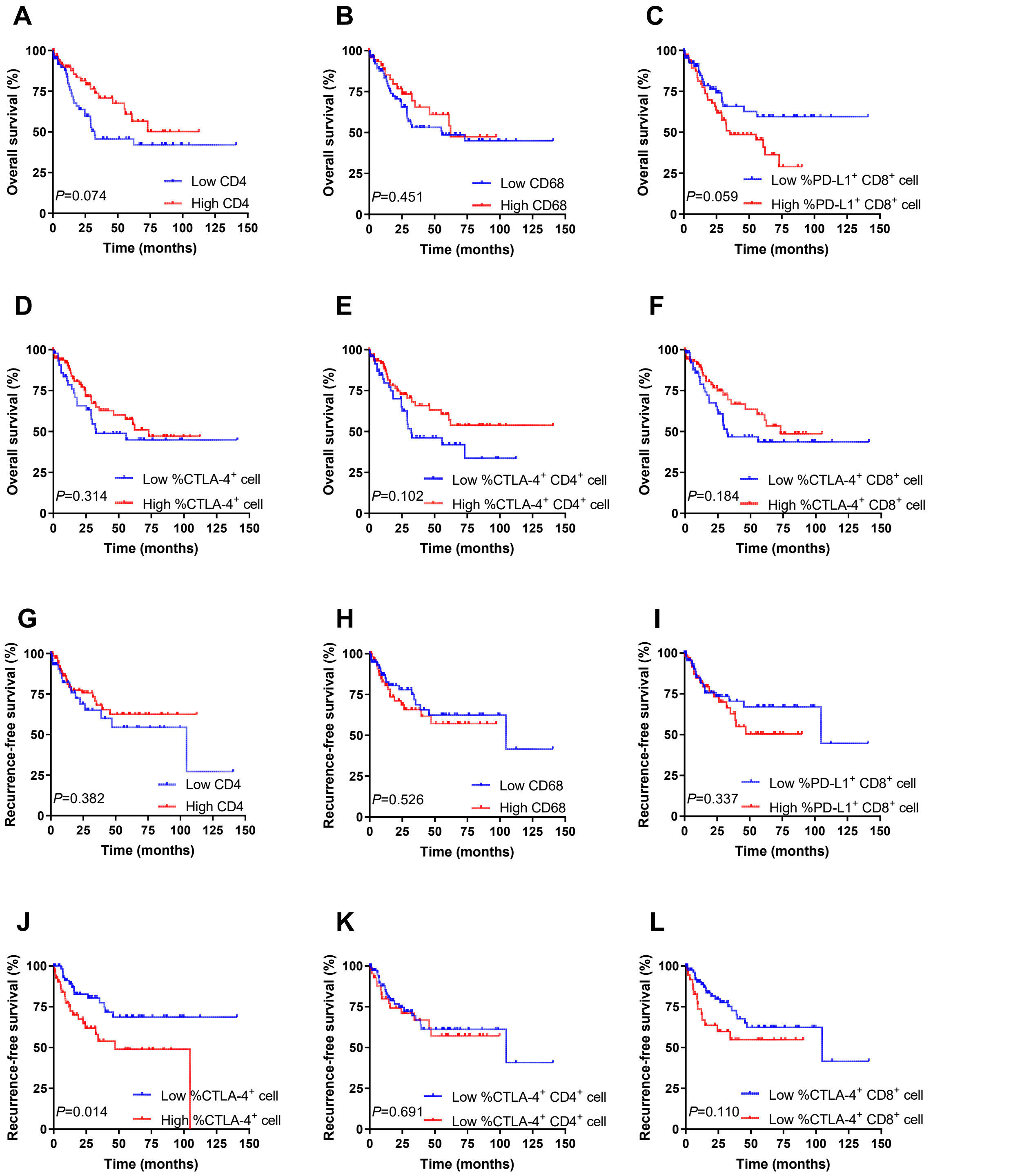

Fig. S5 The prognostic value of immune markers in primary SCLC. (A-F) The prognostic value of immune cell infiltration and checkpoints expression for overall survival. **(G-L)** The prognostic value of immune cell infiltration and checkpoints expression for recurrence-free survival.

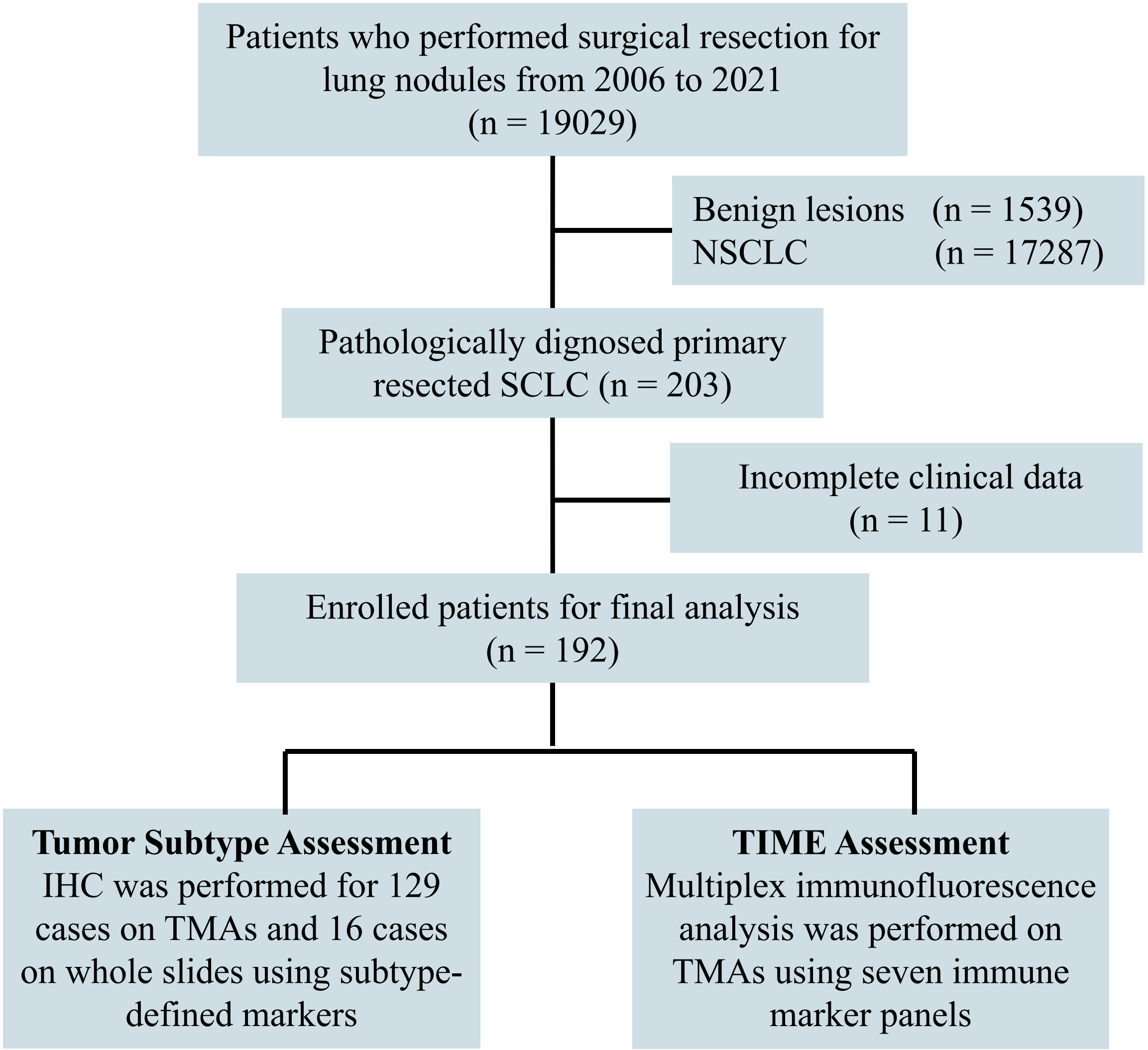

Fig. S6 The Flowchart of the current study.

Table S1. Characteristics of patients performing subtype assess (*N*=145).

**Patient characteristics**	**SCLC**	
	N	**%**
Age		
<60 year	51	35.2
≥60 year	94	64.8
Gender		
Female	23	15.9
Male	122	84.1
Smoking history		
Never	33	22.8
Ever	112	77.2
pTNM		
I	61	42.1
II	36	24.8
III	48	33.1
LVI		
Absent	74	51.0
Present	59	40.7
Unknown	12	8.3
VPI		
Absent	112	77.2
Present	26	18.0
Unknown	7	4.8
Histology type		
Pure	98	67.6
Combined	47	32.4
Adjuvant chemotherapy		
No	38	26.2
Yes	107	73.8

SCLC, small cell lung cancer; LVI, lymphovascular invasion; VPI, visceral pleural invasion;
Unknown: data was not available.

Table S2. The consistency rate of molecular subtype classification between multiple cores of SCLC.

Molecular subtype classification	Number of cases
Consistent	124
Inconsistent	5
Consistency Rate	96.1%

SCLC, small cell lung cancer;

Table S3. Association of SCLC molecular subtypes with patient characteristics.

Patient characteristics	Dominant Subtype (*n*=141)								*P* value
	ASCL1		NEUROD1		POU2F3		VIM		
	n	%	*n*	%	*n*	%	*n*	%	
Gender									0.621
Female	14	15.7	6	21.4	1	11.1	1	6.7	
Male	75	84.3	22	78.6	8	88.9	14	93.3	
Age									0.741
<60year	35	39.3	12	42.9	4	44.4	4	26.7	
≥60year	54	60.7	16	57.1	5	55.6	11	73.3	
Smoking history									0.616
Never	18	20.2	9	32.1	2	22.2	3	20.0	
Ever	71	79.8	19	67.9	7	77.8	12	80.0	
pTNMs									0.867
I	36	40.4	13	46.4	3	33.3	7	46.7	
II-III	53	59.6	15	53.6	6	66.7	8	53.3	
*LVI									0.844
Absent	43	54.4	17	64.3	4	50.0	9	64.3	
Present	36	45.6	11	39.3	4	50.0	5	35.7	
*VPI									0.497
Absent	67	78.8	24	85.7	5	71.4	13	92.9	
Present	18	21.2	4	14.3	2	28.6	1	7.1	
Histology type									0.103
Pure	59	66.3	22	78.6	9	100	8	53.3	
Combined	30	33.7	6	21.4	0	0	7	46.7	
Adjuvant chemotherapy									0.390
No	26	29.2	4	14.3	3	33.3	5	33.3	
Yes	63	70.8	24	85.7	6	66.7	10	66.7	

SCLC, small cell lung cancer; LVI, lymphovascular invasion; VPI, visceral pleural invasion;
*Patients with available data were included in evaluation.

Table S4. The cutoff points of immune markers.

No.	Markers	Cutoff point for OS	Cutoff point for RFS
1	$CD4^+$ T cell/mm^2	148	82
2	$CD8^+$ T cell/mm^2	41	41
3	$CD68^+$ cell/mm^2	1625	1319
4	%PD-$L1^+$ tumor cell	*10.0%	*10.0%
5	%PD-$L1^+$ $CD8^+$ cell	8.0%	10.0%
6	%CTLA-4^+ cell	1.0%	3.3%
7	%CTLA-4^+ $CD4^+$ cell	8.7%	16.2%
8	%CTLA-4^+ $CD8^+$ cell	2.0%	7.1%

*The cutoff point of %PD-$L1^+$ tumor cell was based on clinical practice but not x-tile.
